# Predicting COVID-19 and Respiratory Illness: Results of the 2022–2023 Armed Forces Health Surveillance Division Forecasting Challenge

**Published:** 2024-05-20

**Authors:** Mark L. Bova, Sasha A. McGee, Kathleen R. Elliott, Juan I. Ubiera

**Affiliations:** 1 Armed Forces Health Surveillance Division, Integrated Biosurveillance Branch, Silver Spring, MD

## Abstract

Since 2019, the Integrated Biosurveillance Branch of the Armed Forces Health Surveillance Division has conducted an annual forecasting challenge during influenza season to predict short-term respiratory disease activity among Military Health System beneficiaries. Weekly case and encounter observed data were used to generate 1- through 4-week advanced forecasts of disease activity. To create unified combinations of model inputs for evaluation across multiple spatial resolutions, 8 individual models were used to calculate 3 ensemble models. The accuracy of forecasts compared to the observed activity for each model was evaluated by calculating a weighted interval score. Weekly 1- through 4-week ahead forecasts for each ensemble model were generally higher than observed data, especially during periods of peak activity, with peaks in forecasted activity occurring later than observed peaks. The larger the forecasting horizon, the more pronounced the gap between forecasted peak and observed peak. The results showed that several models accurately predicted COVID-19 cases and respiratory encounters with enough lead time for public health response by senior leaders.

**What are the new findings?:**

By testing a large number of traditional (e.g., ARIMA, EWMA) and non-traditional (e.g., Random Forest, Count Regression) models, this forecasting study improved understanding of which model types were the most accurate and demonstrated a more robust ensemble prediction. The ensemble models developed by the forecasting challenge provided more accurate forecasts in general, when compared to most individual models.

**What is the impact on readiness and force health protection?:**

Respiratory diseases represent a major impediment to military readiness and force health, including interruptions in duties caused by isolation or quarantine requirements as well as morbidity caused by illnesses themselves. Respiratory disease forecasting is a useful tool for senior leaders’ preparations for illness surges.

## 1 BACKGROUND

Seasonal respiratory infections, including influenza and COVID-19, represent a major impediment to military readiness. Accurate forecasts of the burden of respiratory illness in the Department of Defense (DOD) population are crucial for allowing military leaders and public health practitioners to anticipate increases in disease activity and implement preventive measures.

Since 2013, the U.S. Centers for Disease Control and Prevention (CDC) has conducted an annual influenza forecasting challenge, inviting modelers to submit weekly forecasts of influenza-like illness (ILI) or confirmed influenza hospitalizations.^1^ To produce more consistent and reliable forecasts across varying spatial resolutions, forecasting challenges often combine inputs from multiple models into one unified ensemble.^2^

Since 2019, the Integrated Biosurveillance (IB) Branch of the Armed Forces Health Surveillance Division (AFHSD),^3^ part of the Defense Health Agency’s Public Health Directorate, has conducted its own annual forecasting challenge during the influenza season, modeled after that of the CDC. The goal is to predict short-term (1-4 weeks ahead) respiratory disease activity among Military Health System (MHS) beneficiaries within collections of geographically-aligned military installations and medical facilities in the U.S. (“markets”) to support timely decision-making by senior leaders. In addition to forecasting disease activity among MHS beneficiaries, AFHSD also forecasts activity among civilians living in counties within 30 miles of a market. This challenge is open to forecasts submitted by government, academic, and industry partners.

During influenza season, AFHSD-IB reports forecast data through weekly biosurveillance products emailed to more than 3,000 individuals. Stakeholders can access these data as needed to inform resource allocation and prevention activities via an interactive dashboard (by Common Access Card only) updated weekly by AFHSDIB.^4^ This dashboard includes summary information about respiratory illness in each market and DHA network, as well as maps and time series plots of 1- through 4-week ahead forecasts.

This report summarizes the results and lessons from AFHSD’s forecasts for the 2022-2023 forecasting season.

## 2 METHODS

Influenza seasons were defined as epidemiological weeks 40 through 20 according to CDC’s Morbidity and Mortality Weekly Report (MMWR) epidemiological weeks.^5^ The 2022-2023 influenza season began on October 2, 2022 and ended May 20, 2023. The 2022-2023 challenge focused on MHS and civilian COVID-19 cases, as well as MHS COVID-like illness (CLI), ILI, and COVID-19 outpatient encounters.

Weekly respiratory illness data from multiple sources were downloaded for the 2022-2023 influenza season. MHS COVID-19 cases were collected by AFHSD’s Epidemiology & Analysis Branch using laboratory and reportable medical event (RME) data provided by the Defense Centers for Public Health (DCPH)–Portsmouth and DCPH–Aberdeen. The Armed Forces RME Guidelines and Case Definitions document defines 70 DOD RMEs, which closely mirror the nationally notifiable diseases monitored by CDC.^6,7^ A confirmed case of COVID-19 in MHS beneficiaries was defined using laboratory, clinical, epidemiological, and death certificate data (Unpublished, **Supplementary Table 1**). Civilian COVID-19 cases, by county, were obtained from HHS Protect and defined according to CDC criteria.^8,9^ MHS outpatient encounters were extracted from DOD’s Electronic Surveillance System for the Early Notification of Community-based Epidemics (ESSENCE). CLI, ILI, and COVID-19 encounter case definitions were developed internally using International Classification of Diseases, 10th Revision, Clinical Modification (ICD-10-CM) diagnosis codes, and are provided in **Supplementary Table 1**.

Weekly case and encounter observed data were used to generate 1- through 4-week ahead forecasts of disease activity. Forecasts were generated using various models, including time series (including Autoregressive Integrated Moving Average [ARIMA], Error, Trend, Seasonal [ETS], Exponentially Weighted Moving Average [EWMA], and Vector Autoregressive [VAR]), machine learning (including Random Forest), and count regression (including Poisson, Negative Binomial, and Log-binomial) models. To create unified combinations of model inputs for evaluation across multiple spatial resolutions,^9^ individual models were used to calculate the 3 ensemble models: 1) the average of the time series and machine learning models–ENSEMBLE, 2) the average of the 3 best-performing time series and machine learning models–ENSEMBLE_TOP, and 3) the average of the count regression models–ENSEMBLE_CNT.

The accuracy of forecasts compared to the observed activity for each model was evaluated by calculating a weighted interval score (WIS),^10^ a metric also used by the CDC, that compares performance among models. A lower score indicates better model performance. All analyses were conducted using R software (version 4.1, The R Foundation for Statistical Computing, Vienna, Austria). The R packages “fable,” “randomForest,” and “tscount” were used to generate forecasts and the “evalcast” package to calculate the WIS.^11,12,13,14^

## 3 RESULTS

**Figure 1:**
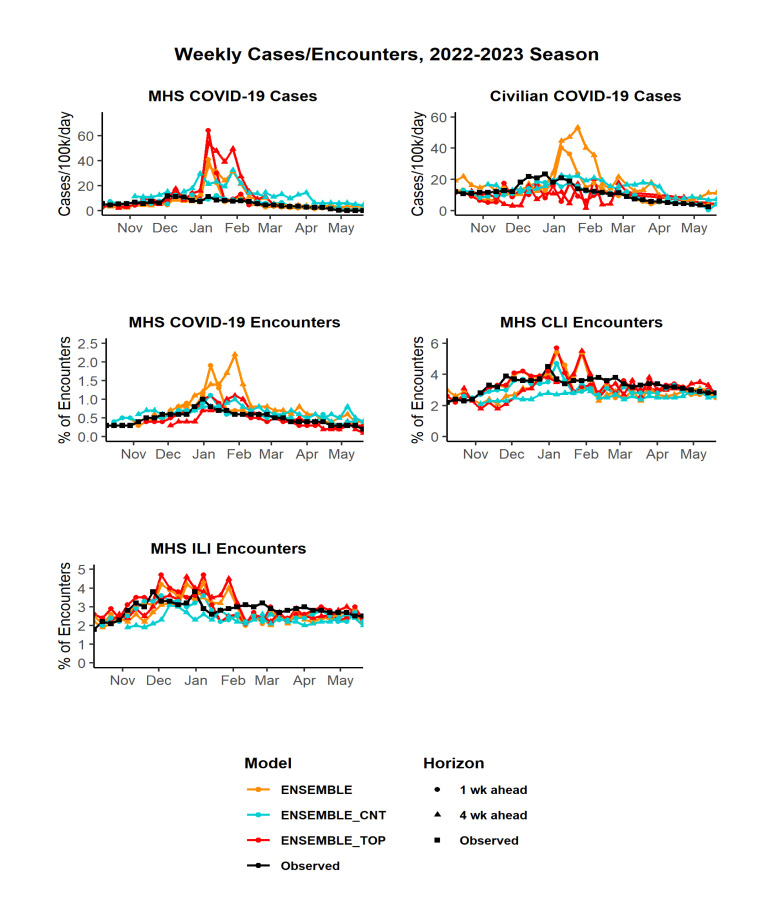
Weekly Forecasts Versus Observed Data by Ensemble Model and Forecasting Horizon, All U.S. Surveillance Markets, October 2022-June 2023

Weekly observed counts of MHS and civilian COVID-19 cases by market were converted to population-adjusted rates, while weekly observed MHS outpatient encounters were converted to a percentage of total outpatient encounters for that week. Weekly 1- through 4-week ahead forecasts for each ensemble model were generally higher than observed data, especially during periods of peak activity (December through February), with peaks in forecasted activity occurring later than observed peaks (**Figure1**). The larger the forecasting horizon (i.e., 4 weeks ahead versus 1 week), the more pronounced the gap between forecasted peak and observed peak.

Forecasts of peak MHS COVID-19 case rates were mostly higher than observed, ranging from 44% higher for the ENSEMBLE_CNT model to 457% higher for the ENSEMBLE_TOP model (**Table 1**). Peak civilian COVID-19 case rate forecasts were more accurate, ranging from 13% lower (ENSEMBLE_CNT) to 99% higher (ENSEMBLE). Peak encounter forecasts for the ENSEMBLE_CNT model were lower than observed peaks (16% and 9% lower for ILI and CLI, respectively) and equal to the observed peak for COVID-19 encounters. Peak encounter forecasts for the ENSEMBLE_TOP model were higher than observed peaks, including 24% higher for ILI, 27% higher for CLI, and 10% higher for COVID-19 encounters. Peak week forecasts tended to be 2 to 6 weeks later than observed for most ensemble models and forecast targets. The ENSEMBLE_CNT model accurately predicted forecasts of peak civilian COVID-19 cases and MHS ILI encounters, however.

**Figure 2:**
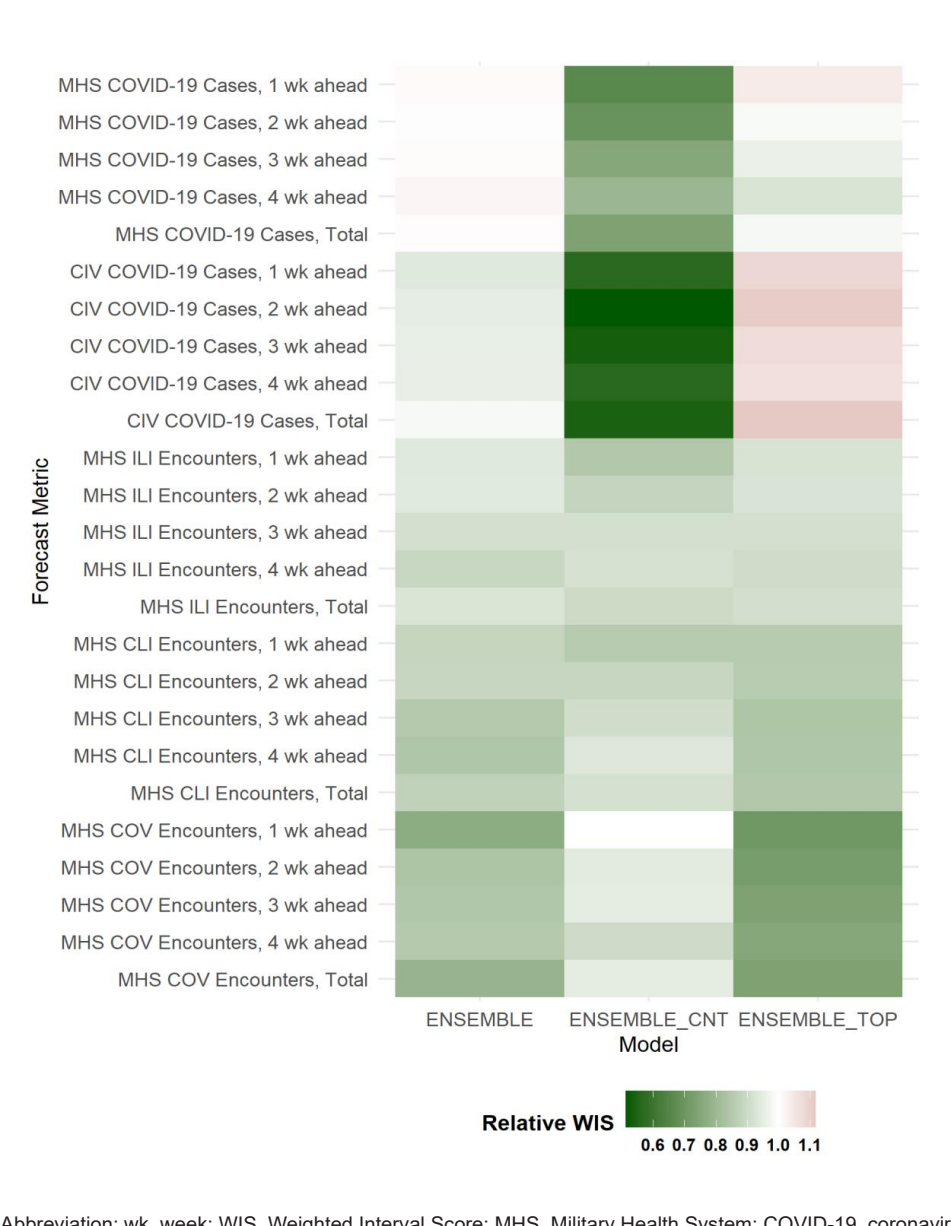
Assessment of Error in the Ensemble Models by Forecasting Target and Horizon Based on Median WIS, All U.S. Surveillance Markets

Overall, the ENSEMBLE_CNT model had the lowest WIS of all forecasting horizons, indicating the most accurate forecasts for civilian and MHS COVID-19 cases (**Figure2**). The ENSEMBLE_TOP model was the most accurate for COVID-19 encounter forecasts, while all 3 ensemble models performed similarly for CLI and ILI encounters. Model performance decreased as forecast horizons increased, with the median WIS for all 4-week ahead forecasts of the ensemble models increasing between 10% (MHS ILI encounters) and 98% (civilian COVID-19 cases) compared to 1-week ahead forecasts.

## 4 DISCUSSION

This is the first published results from the AFHSD Respiratory Forecasting Challenge since it was begun in 2019. Respiratory disease forecasting was more challenging during the 2022-2023 influenza season, due in part to decreased COVID-19 activity compared to prior years and ILI resurgence (**Supplementary Table 2**). Peak observed MHS and civilian COVID-19 case rates in 2022-2023 were 95% and 91% lower, respectively, compared to the 2021-2022 season, while peak observed MHS COVID-19 and CLI encounters were 76% and 26% lower, respectively, than the prior season. Conversely, peak observed MHS ILI encounters during the 2022-2023 season were 41% higher than during the 2021-2022 season and 111% higher than during the 2020-2021 season. Historical data for the previous 2 seasons were, therefore, not predictive of respiratory activity in 2022-2023.

Ensemble models generally provided more accurate forecasts, especially the ENSEMBLE_CNT and ENSEMBLE models, compared to most individual models (**Supplementary Figure**). Although certain individual models outperformed ensemble models for specific forecasting targets, including the Random Forest model for MHS COVID-19 case forecasts and the Poisson model for civilian COVID-19 case forecasts, each performed similarly when compared to the best-performing ensemble model. Model performance decreased as the forecasting horizon increased, with WIS scores ranging from 10% to 95% higher on average for 4-week ahead forecasts compared to 1-week ahead forecasts. These results are consistent with a previous publication of COVID-19 forecasts in the U.S. COVID-19 Forecast Hub that found that an ensemble model comprised of 27 individual models was consistently more accurate than the individual models, and that the accuracy of forecasting models decreased as forecast horizons increased.^15^

This forecasting study has several strengths. First, the forecasting results showed that several models accurately predicted COVID-19 cases and respiratory encounters with enough lead time for senior leaders to take action. Second, this forecasting study tested a large number of traditional (e.g., ARIMA, EWMA) and non-traditional (e.g., Random Forest, Count Regression) models, increasing our understanding of which types of models were most accurate and providing a more robust ensemble prediction.

The forecasting of the 2022-2023 season also showed several limitations that may have affected model accuracy. COVID-19 cases may have been generally under-reported due to the large number of asymptomatic cases and use of at-home testing, both within DOD and civilian populations. Data reporting schedules, particularly for civilian COVID-19 cases, changed dramatically during the season after the May 11, 2023 end of the U.S. Public Health Emergency for COVID-19. This policy change disrupted county case reporting by CDC.^16^ Many states and military treatment facilities also changed their COVID-19 case reporting schedules, from daily to weekly, monthly, or not at all. To abridge some of gaps in COVID-19 reporting, health encounter data from DOD ESSENCE could be utilized, but syndromic surveillance systems such as ESSENCE may suffer from inconsistent data quality between reporting sites and gaps in coverage.^17^ In addition, these data can also lag by at least 4 days from the encounter date, leading to under-reporting of health encounters during the most recent week; these data present challenges for forecasting, as the observed value for this week may change significantly in subsequent weeks. During the 2022-2023 season, reported numbers of civilian and MHS COVID-19 cases for a given week increased by as much as 50% 1 month after an initial reporting date, as older cases were reported, while MHS encounter data ranged from a 40% decrease to a 40% increase as additional encounters populated the system. Efforts were made to account for potential backfill in each market for both case and encounter data prior to generating weekly forecasts, but forecasting analysis can be challenging due to unpredictable data processing schedules. Other limitations included the availability and usefulness of covariate data. Data that previously relied on for COVID-19 forecasting, including vaccination and case data, became less reliable or unavailable during the season.

Another limitation of this study is the relative usefulness and timeliness of the forecasts. As mentioned, forecast accuracy decreased as forecasting horizon increased. The data lags in ESSENCE, compounded by the time constraints of downloading and aggregating weekly data and generating weekly forecasts, meant that weekly forecasts were not available for senior leaders until nearly 1 week after the most recently observed data. This circumstance renders the 1-week ahead forecasts of disease activity mostly unusable, limiting senior leaders’ response time to 2-week ahead forecasts. Although the 3- and 4-week ahead forecasts provide adequate time for senior leaders to make necessary preparations, their accuracy is greatly diminished compared to 1- and 2-week ahead forecasts. Efforts to improve the utility of 1- and 2-week ahead forecasts may be achieved by downloading data earlier each week and generating weekly forecasts more efficiently, but efforts for improving the more distant horizon forecasts and expanding beyond 4 weeks are current priorities.

Future AFHSD-IB respiratory forecasting challenges will consider additional covariates such as environmental data and combine time series and count regression forecasts into a single ensemble model. The incorporation of new models, such as neural network models, machine learning models, and wavelet forecasting, will also be explored. More emphasis will be placed on non-pandemic seasons to lessen the impacts of changes in COVID-19 and influenza reporting. Forecasting will focus on more consistently available data sources for both DOD and civilian populations, including COVID-19 hospitalizations, influenza hospitalizations, and health encounter data. As the time elapsed since the initial years of the COVID-19 pandemic increases, historical data may become more reliable in predicting the volume and peak activity for COVID-19 and other respiratory diseases during upcoming influenza seasons.

## Figures and Tables

**Supplementary Table 1:** AFHSD Forecasting Target Definitions

Forecasting Target	Case Definition	
MHS COVID-19 Case	Confirmed: Detection of SARS-CoV-2 nucleic acid (RNA) by molecular amplification from a clinical or autopsy specimen
	Probable: Meets any of the following criteria:
	1) Epidemiologically linked to another case of COVID-19 with no confirmatory COVID-19 laboratory testing and meets the following clinical description of a case:
	a. At least TWO of the following symptoms: fever, chills, rigors, myalgia, headache, sore throat, nausea or vomiting, diarrhea, fatigue, congestion or runny nose
	OR
	b. Any ONE of the following symptoms: cough, shortness of breath, difficulty breathing, new olfactory disorder, or new taste disorder
	OR
	c. Severe respiratory illness with at least 1 of the following: clinical or radiographic evidence of pneumonia, or acute respiratory distress syndrome (ARDS)
	2) Detection of SARS-CoV-2 antigen from a respiratory specimen
	3) A death certificate that lists COVID-19 disease or SARS-CoV-2 as an underlying cause of death or a significant condition contributing to death with no confirmatory COVID-19 laboratory testing
MHS CLI Encounter	Any of the following ICD-10-CM diagnosis codes in any diagnostic position: B34.2, B97.21, B97.29, J00, J06.9, J12.81, J12.89, J12.9, J16.8, J17, J18.0, J18.1, J18.8, J18.9, J20.8, J20.9, J22, J40, J80, R05, R05.9, R06.0, R06.00, R06.02, R06.03, R06.09, U07.1, R43.0, R43.2, U07.1, J84.111
MHS ILI Encounter	Any of the following ICD-10-CM diagnosis codes in any diagnostic position: B97.89, H66.9, H66.90, H66.91, H66.92, H66.93, J00, J01.9, J01.90, J06.9, J09, J09.X, J09.X1, J09.X2, J09.X3, J09.X9, J10, J10.0, J10.00, J10.01, J10.08, J10.1, J10.2, J10.8, J10.81, J10.82, J10.83, J10.89, J11, J11.0, J11.00, J11.08, J11.1, J11.2, J11.8, J11.81, J11.82, J11.83, J11.89, J12.89, J12.9, J18, J18.1, J18.8, J20.9, J22, J40, R05, R05.9
MHS COVID-19 Encounter	Any of the following ICD-9-CM or ICD-10-CM diagnosis codes in any diagnostic position: B97.29, U07.1, Z03.818, Z20.828, B34.2, J12.81, 079.82, 480.3, V01.82

Abbreviations: MHS, Military Health System; COVID-19 coronavirus disease 2019; CLI, COVID-like illness; ILI, influenza-like illness

**Table 1:** Comparison of Observed and Forecasted Activity by Forecast Target, All U.S. Surveillance Markets, 2-Week Forecasting Horizon

Forecast Target	Peak Activity				Peak Week			
	Observed Activity	Forecasted Activity			Observed Week	Difference Between Forecasted and Observed Week		
		ENSEMBLE	ENSEMBLE_TOP	ENSEMBLE_CNT		ENSEMBLE	ENSEMBLE_TOP	ENSEMBLE_CNT
MHS COVID-19 cases 100k/day	11.6	40.8	64.6	16.7	48	+6	+6	+2
Civilian COVID-19 cases 100k/day	23.3	46.3	18.9	20.3	51	+2	+3	0
MHS % ILI encounters	3.8%	4.2%	4.7%	3.2%	47	+2	+2	0
MHS % CLI encounters	4.5%	5.3%	5.7%	4.1%	52	+2	+2	+2
MHS % COVID-19 encounters	1.0%	2.0%	1.1%	1.0%	52	+2	+2	+2

Abbreviations: MHS, Military Health System; COVID-19, coronavirus disease 2019; k, 1,000; CLI, COVID-like illness; ILI, influenza-like illness.

COVID-19 case definition: Any positive laboratory result for SARS-CoV-2 or a confirmed COVID-19 reportable medical event

CLI encounter definition: Any of the following ICD-10 diagnosis codes in any diagnostic position: B34.2, B97.21, B97.29, J00, J06.9, J12.81, J12.89, J12.9, J16.8, J17, J18.0, J18.1, J18.8, J18.9, J20.8, J20.9, J22, J40, J80, R05, R05.9, R06.0, R06.00, R06.02, R06.03, R06.09, U07.1, R43.0, R43.2, U07.1, J84.111

ILI encounter definition: Any of the following ICD-10 diagnosis codes in any diagnostic position: B97.89, H66.9, H66.90, H66.91, H66.92, H66.93, J00, J01.9, J01.90, J06.9, J09, J09.X, J09.X1, J09.X2, J09.X3, J09.X9, J10, J10.0, J10.00, J10.01, J10.08, J10.1, J10.2, J10.8, J10.81, J10.82, J10.83, J10.89, J11, J11.0, J11.00, J11.08, J11.1, J11.2, J11.8, J11.81, J11.82, J11.83, J11.89, J12.89, J12.9, J18, J18.1, J18.8, J20.9, J22, J40, R05, R05.9

COVID-19 encounter definition: Any of the following ICD-9 or ICD-10 diagnosis codes in any diagnostic position: B97.29, U07.1, Z03.818, Z20.828, B34.2, J12.81, 079.82, 480.3, V01.82

**Supplementary Table 2:** Comparison of Observed Activity by Influenza Season, All U.S. Surveillance Markets

Forecast Target	Peak Activity				Peak Week			
	2022-2023	2021-2022	2020-2021	2019-2020	2022-2023	2021-2022	2020-2021	2019-2020
MHS COVID-19 cases	11.6	216.7	47.0	NA	48	1	1	NA
Civilian COVID-19 cases	23.3	264.0	79.4	NA	51	2	1	NA
MHS ILI encounters	3.8%	2.7%	1.8%	5.3%	47	52	46	52
MHS CLI encounters	4.5%	6.1%	3.6%	3.9%	52	52	52	1
MHS COVID-19 encounters	1.0%	4.2%	4.1%	NA	52	52	1	NA

COVID-19 case definition: Any positive laboratory result for SARS-CoV-2 or a confirmed COVID-19 reportable medical event

CLI encounter definition: Any of the following ICD-10 diagnosis codes in any diagnostic position: B34.2, B97.21, B97.29, J00, J06.9, J12.81, J12.89, J12.9, J16.8, J17, J18.0, J18.1, J18.8, J18.9, J20.8, J20.9, J22, J40, J80, R05, R05.9, R06.0, R06.00, R06.02, R06.03, R06.09, U07.1, R43.0, R43.2, U07.1, J84.111

ILI encounter definition: Any of the following ICD-10 diagnosis codes in any diagnostic position: B97.89, H66.9, H66.90, H66.91, H66.92, H66.93, J00, J01.9, J01.90, J06.9, J09, J09.X, J09.X1, J09.X2, J09.X3, J09.X9, J10, J10.0, J10.00, J10.01, J10.08, J10.1, J10.2, J10.8, J10.81, J10.82, J10.83, J10.89, J11, J11.0, J11.00, J11.08, J11.1, J11.2, J11.8, J11.81, J11.82, J11.83, J11.89, J12.89, J12.9, J18, J18.1, J18.8, J20.9, J22, J40, R05, R05.9

COVID-19 encounter definition: Any of the following ICD-9 or ICD-10 diagnosis codes in any diagnostic position: B97.29, U07.1, Z03.818, Z20.828, B34.2, J12.81, 079.82, 480.3, V01.82

Abbreviations: MHS, Military Health System; COVID-19, coronavirus disease 2019; k, 1,000; CLI, COVID-like illness; ILI, influenza-like illness; NA, not applicable.

**Supplementary Figure:**
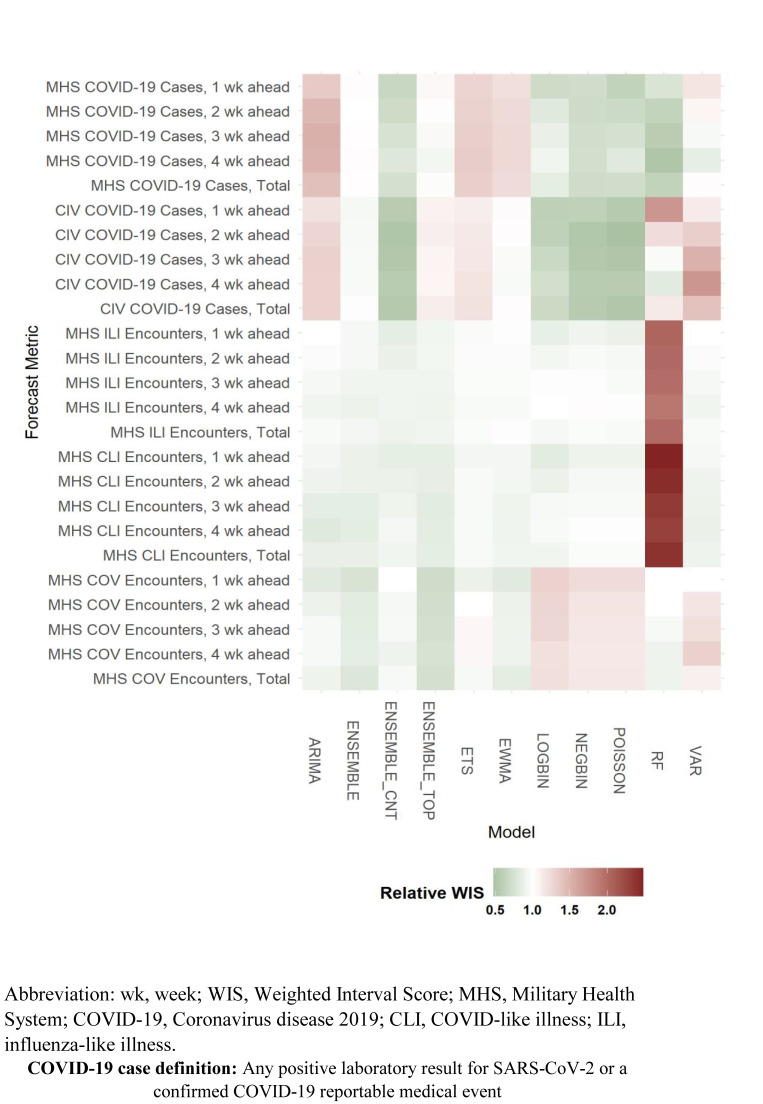
Assessment of Error in Forecasting Models by Forecasting Target and Horizon Based on Relative Median WIS, All U.S. Surveillance Markets

## AUTHORS' AFFILIATION

Armed Forces Health Surveillance Division, Integrated Biosurveillance Branch, Silver Spring, MD: Mr. Bova, Dr. McGee, Ms. Elliott, Mr. Ubiera
